# Integrated network pharmacology and bioinformatics to identify therapeutic targets and molecular mechanisms of Huangkui Lianchang Decoction for ulcerative colitis treatment

**DOI:** 10.1186/s12906-024-04590-3

**Published:** 2024-07-23

**Authors:** Zongqi He, Xiang Xu, Yugen Chen, Yuyu Huang, Bensheng Wu, Zhizhong Xu, Jun Du, Qing Zhou, Xudong Cheng

**Affiliations:** 1 Kunshan Hospital of Chinese Medicine, Kunshan, 215300 PR China; 2 Zhangjiagang TCM Hospital Affiliated to Nanjing University of Chinese Medicine, Zhangjiagang, 215600 PR China; 3 Department of Colorectal Surgery, Affiliated Hospital of Nanjing University of Chinese Medicine, No. 155, Hanzhong Road, Nanjing, Jiangsu Province 210004 PR China; 4 Pharmacy Department, Suzhou TCM Hospital Affiliated to Nanjing University of Chinese Medicine, No. 18, Yang Su Road, Suzhou, Jiangsu Province 215009 PR China

**Keywords:** Huangkui Lianchang Decoction, Ulcerative colitis, Network pharmacology, Bioinformatics, TLR4/MyD88/NF-κB signaling pathway

## Abstract

**Background:**

Huangkui Lianchang Decoction (HLD) is a traditional Chinese herbal formula for treating ulcerative colitis (UC). However, its mechanism of action remains poorly understood. The Study aims to validate the therapeutic effect of HLD on UC and its mechanism by integrating network pharmacology, bioinformatics, and experimental validation.

**Methods:**

UC targets were collected by databases and GSE19101. The active ingredients in HLD were detected by ultra-performance liquid chromatography-tandem mass spectrometry. PubChem collected targets of active ingredients. Protein–protein interaction (PPI) networks were established with UC-related targets. Gene Ontology and Kyoto Encyclopedia (KEGG) of Genes and Genomes enrichment were analyzed for the mechanism of HLD treatment of UC and validated by the signaling pathways of HLD. Effects of HLD on UC were verified using dextran sulfate sodium (DDS)-induced UC mice experiments.

**Results:**

A total of 1883 UC-related targets were obtained from the GSE10191 dataset, 1589 from the database, and 1313 matching HLD-related targets, for a total of 94 key targets. Combined with PPI, GO, and KEGG network analyses, the signaling pathways were enriched to obtain IL-17, Toll-like receptor, NF-κB, and tumor necrosis factor signaling pathways. In animal experiments, HLD improved the inflammatory response of UC and reduced UC-induced pro-inflammatory factors such as Tumor Necrosis Factor Alpha (TNF-α), interleukin 1β (IL-1β), and interleukin 6 (IL-6). HLD suppressed proteins TLR4, MyD88, and NF-κB expression.

**Conclusions:**

This study systematically dissected the molecular mechanism of HLD for the treatment of UC using a network pharmacology approach. Further animal verification experiments revealed that HLD inhibited inflammatory responses and improved intestinal barrier function through the TLR4/MyD88/NF-κB pathway.

**Supplementary Information:**

The online version contains supplementary material available at 10.1186/s12906-024-04590-3.

## Background

Ulcerative Colitis (UC) is a group of persistent and recurrent inflammatory conditions affecting the intestine and is classified as an inflammatory bowel disease (IBD), along with Crohn's disease [1, 2]. UC develops in the innermost mucosal layer of the distal gastrointestinal tract continuously, usually starting in the rectum and spreading into the colon. The incidence and prevalence of UC are increasing globally, including in developing regions and Asian countries [3, 4]. Despite extensive research, the etiology of UC is still not fully understood. UC may be caused by multiple factors, such as excessive inflammation, genetics, environmental influences, diet, and gut microbiome imbalances [2]. As the pathogenesis of UC is not well understood, it is currently incurable. Common treatments for UC include 5-ASA, steroids, immunosuppressants, and targeted molecular drugs, which vary in use depending on the severity and extent of the disease [2, 5]. These treatments help control the inflammatory response in the intestine; however, they are ineffective for a significant proportion of patients and can also cause serious adverse effects. Therefore, an effective and safe alternative treatment is required.

An increasing amount of literature supports the efficacy of traditional Chinese herbal medicine in the treatment of UC, with minimal adverse effects [6–10]. Huangkui Lianchang Decoction (HLD) is a traditional Chinese medicinal preparation made from a combination of six herbs. Its concentrated aqueous solution has been used for many years in the clinical treatment of UC. HLD has been used on its own to treat mild to moderate E1 and E2 UC, or as an additional treatment for moderate to severe UC, and has shown good clinical effectiveness. In a previous clinical observation, HLD enema was found to be effective in treating distal UC by significantly improving clinical symptoms, suppressing the inflammatory response, and improving quality of life [11]. Another retrospective clinical study found that the addition of HLD enema to the treatment regimen was more effective in controlling clinical symptoms and promoting mucosal healing for patients with mild to moderate UC [12]. In our previous study, we applied HLD to treat a mouse model of DSS-induced UC and found that HLD reduced the DAI index and attenuated colonic tissue damage, possibly through inhibition of the NF-κB pathway for anti-inflammatory effects [13]. Immediately after that, we verified that HLD alleviated intestinal inflammation by inhibiting the NF-κB pathway and autophagy in vivo and in vitro experiments, but there was no interaction between the NF-κB pathway and autophagy [14]. The above results suggest that HLD can exert anti-inflammatory effects via the NF-κB pathway in the DSS-induced UC mouse model, but the mechanism of the HLD-regulated NF-κB pathway remains unclear.

Traditional herbal medicines are often prepared as aqueous extracts containing active ingredients that act on multiple targets [15–17]. HLD contains six herbs and a complex composition of thousands of active ingredients in its aqueous extract. In addition to the known NF-κB and autophagy pathways, other potential targets of HLD are unknown. By utilizing “compound-protein/gene-disease” networks, network pharmacology is capable of uncovering the regulatory principles of small molecules in a high-throughput manner, which provides significant advantages for complex system analysis [17]. The combination of network pharmacology, bioinformatics, and experimental validation has become a useful approach for identifying targets and mechanisms. Several studies [16, 17] have utilized network pharmacology approaches to investigate the mechanisms of Huai Hua San, and Wumei Wan, respectively, for the treatment of UC. These investigations suggest that network pharmacology can be a useful tool for exploring the potential mechanisms of HLD for UC treatment.

In this study, we used ultra-performance liquid chromatography-tandem mass spectrometry (UPLC-MS/MS) to analyze the active components of HLD in an aqueous solution. A combination of network pharmacology, bioinformatics, and molecular docking was employed to identify possible targets of HLD for UC treatment. Finally, we validated the effect of HLD on UC by demonstrating its ability to alleviate dextran sulfate sodium (DSS)-induced colitis in UC mice by inhibiting the TLR4/MyD88/NF-κB signaling pathway in animal experiments. The findings of this study provide new scientific evidence for the prevention and treatment of UC with HLD.

## Methods

### Drugs and reagents

HLD is composed of Abelmoschus manihot (*Abelmoschus manihot* (L.) Medik), Herba Euphorbiae humifusae (*Euphorbia humifusa* Willd), Herba Pteridis multifidae (*Pteris multifida* Poir), Radix Arnebiae seu Lithospermi (*Arnebia euchroma* I.M. Johnst), Radix Rubiae (*Rubia cordifolia* L.), and Galla Chinensis (*Rhus chinensis* Mill). The plant name has been checked with http://www.worldfloraonline.org. Experimental research on plants complies with the IUCN Policy Statement on Research Involving Species at Risk of Extinction and the Convention on the Trade in Endangered Species of Wild Fauna and Flora. For this study, the herbs were provided by Suzhou Tianling Chinese Herbal Medicine Co., Ltd. All herbs complied with the Chinese Pharmacopoeia or corresponding herbal standards. Mesalazine enemas (Vifor AG Zweigniederlassung Medichemie Ettingen, Switzerland) were purchased from Kangzhe Pharmaceutical Company (Shenzhen, China). DSS was obtained from MP Biomedicals, LLC (Solon, OH, USA). ELISA assay kits for tumor necrosis factor-α (TNF-α), interleukin-10 (IL-10), interleukin-6 (IL-6), and interleukin 1β (IL-1β) were purchased from Thermo Fisher Scientific (San Diego, CA, USA). Antibodies against TLR4 and β-actin were provided by Wuhan Servicebio Technology Co. Ltd. The anti-MyD88 antibody was provided by ProteinTech Group Inc. (Chicago, IL, USA) and the anti-NF-κBp65 antibody was purchased from Cell Signaling Technology (Danvers, MA, USA).

### Preparation of HLD extracts

HLD extracts were prepared according to a previously described method. Briefly, six herbs, Abelmoschus manihot (15 g), Herba Euphorbiae humifusae (15 g), Herba Pteridis multifidae (15 g), Radix Arnebiae seu Lithospermi (7.5 g), Radix Rubiae (7.5 g), and Galla Chinensis (2.5 g) were soaked for 60 min and boiled twice for 40 min in distilled water equivalent to eight times the amount of the herb. The extracts were centrifuged at 3000 rpm for 30 min and concentrated to 2.5 g of raw drug in each milliliter solution. The samples were then subjected to steam sterilization at 100 °C for 30 min and stored at 4 °C for further experiments.

### Compositional analysis of HLD using UPLC-MS/MS

The active compounds in the HLD extracts were characterized using UPLC-MS/MS (Table 1). Briefly, HLD extracts were dissolved in methanol, filtered through a 0.22 μm microporous membrane, and separated using a Waters I CLASS UPLC and an Acquity HSS T3 column (1.8 μm, 2.1 × 100 mm). The compounds were detected using an ABSciex 6500 plus QTRAP mass spectrometer and the mobile phase parameters were set as below at 0.4 mL/min flow rate.

**Table 1:** Table of mobile phase gradient elution in UPLC-MS/MS analysis

Time	Mobile phase A, 0.1% formic acid aqueous solution	Mobile phase B, 0.1% formic acid acetonitrile
0–0.5 min	98%	2%
0.5–10 min	98–50%	2–50%
10–11 min	50–5%	50–95%
11–13 min	5%	95%
13–13.1 min	5–98%	95–2%
13.1–15 min	98%	2%

The column temperature was set to 40 °C. The injection volume was 2.0 μL. Typical mass spectrometry detection parameters were followed, namely an ion spray voltage of + 5500 V, curtain gas: 35 psi, temperature: 400 °C, ion source gas 1: 60 psi, and DP: ± 100 V. Multiple reaction monitoring (MRM) data acquisition and processing were performed using Analyst Workstation software (version 1.6.3, AB SCIEX, MA, USA).

### Network pharmacological analysis

#### Prediction targets of active compounds in HLD

Active compounds identified using UPLC-MS/MS were screened for oral bioavailability (OB) ≥ 30% and drug similarity (DL) ≥ 0.18 were retained for further studies. All chemical structures were imported into Swiss Target Prediction (http://www.swisstargetprediction.ch/), PubChem (https://www.pubchem.ncbi.nlm.nih.gov/), and the ITCM database (http://itcm.biotcm.net/) [18] to predict targets.

#### Acquisition of UC targets

UC targets were obtained from the Gene Expression Omnibus (GEO) and public databases and the microarray expression profiling dataset GSE10191 was downloaded from GEO (http://www.ncbi.nlm.nih.gov/geo/). The dataset is based on the GPL5760 Affymetrix GeneChip Human Genome U133 Plus 2.0 and contains 23 samples, including eight colon tissue samples from UC patients, 11 healthy human tissue samples, and four internal control samples. The R packages (“limma” and “pheatmap”) were used to analyze GSE10191. Differentially Expressed Gene (DEGs) between normal and UC tissues were detected according to a criterion-adjusted *P* value of *P* < 0.05 and |logic|> 1 and visualized using volcano maps.

Targets associated with UC were retrieved by searching for the keyword “ulcerative colitis” in the OMIM (https://omim.org/), GeneCards (https://www.genecards.org/), and PharmGKB (https://www.pharmgkb.org/) databases, as well as the TTD (http://db.idrblab.net/ttd/) and the DisGeNET (https://www.disgenet.org/) platform. All collected disease-related targets were converted into target IDs in the UniProt database ( https://www.uniprot.org/).

#### Construction of protein–protein interaction (PPI) networks

Network construction is important for biological functions and processes as it helps understand the molecular mechanisms of disease and the discovery of novel targets. The R package (“Venn”) mapped VENN and screened for overlapping targets between HLD and UC. These overlapping targets were submitted to the STRING (https://string-db.org/) network for PPI analysis with a minimum required interaction score of ≥ 0.9. Subsequently, PPI results were exported from STRING and imported into Cytoscape 3.8.2 for network construction and visualization. CytoNCA was used to select pivot genes with the top values of degree centrality (DC), betweenness centrality (BC), and closeness centrality (CC).

#### GO function and KEGG pathway enrichment analysis

GO and pathway enrichment analyses were performed using the MetaScape (https://metascape.org) annotation resource. GO enrichment focused on the biological processes, cellular composition, and molecular functions of the targets. After transferring the HLD-UC gene symbols to the relevant Entrez IDs, GO enrichment and KEGG pathway enrichment analyses were performed to further investigate the identified potential anti-UC drugs. UC target genes for HLD were analyzed based on R 4.0.2 and related R packages (“cluster profile,” “ggplot2,” “enrichplot,” “stringi,” “pathview,” “DOSE,” and “org.Hs.eg.db”). Function and pathways with *P* < 0.05 were considered statistically significant and were retained.

#### Construction of chemical-target-pathway network

A chemical-target-pathway network was constructed using Cytoscape_v3.8.2 (https://www.cytoscape.org/). “Nodes” were indicated to the active compounds, targets, and pathways. In addition, “edges” represented the association between one node and another node. The degree of association between nodes was analyzed based on the degree value. After constructing the PPI network, the pivot point genes were screened. By intersecting the active ingredient targets of HLD with the disease targets of UC, we obtained the relevant targets of HLD for the treatment of UC.

#### Molecular docking

To assess the plausibility of the target-compound association and identify novel therapeutic components, molecular docking between the core compound and the core targets was performed. The crystal structures of the core proteins were downloaded from the Protein Data Bank (PDB) (http://www.rcsb.org/pdb). Candidate compounds with two-dimensional structures were downloaded from the PubChem database (https://pubchem.ncbi.nlm.nih.gov/). Molecular docking was performed using Discovery Studio software (BIOVIA; CA, USA). Libdock scores ≥ 90 indicated that the ligand had a strong affinity for the receptor and was more likely to bind. The higher the score, the more stable the binding between the ligand and receptor. Meanwhile, the interactions between the HLD core molecules and the core targets were analyzed, including electrostatic, hydrogen bonding, hydrophobic, and van der Waals interactions.

### Experimental validation

#### Experimental animals

Fifty C57BL/6 male mice (grade SPF) weighing 22 ± 2 g (eight weeks old) were obtained from JOINN Laboratories (Suzhou), Inc. (animal quality certificate number: No.202239726, animal license number: SCXK (Jiangsu) 2018–0006). The animals were housed in an SPF-grade animal room in the central laboratory of Suzhou TCM Hospital affiliated to Nanjing University of Chinese Medicine, at a temperature of 22 ± 2 °C with 60 ± 5% humidity. With seven days of adaptive feeding before experiments. The animal experimental procedures conformed to animal ethics requirements and were approved by the Experimental Animal Ethics Committee of Suzhou TCM Hospital, under Grant No. 2020 Ethical Animal Approval 002. The study was conducted by Directive 2020/63/EU to protect the experimental animals. The study followed the ARRIVE guidelines 2.0 for design, analysis, and reporting.

#### Establishment of mouse ulcerative colitis model

Mice were randomly divided into five groups of 10 mice each: control group, DSS model group, mesalazine group (DSS + Mesalazine), low-dose HLD group (DSS + HLD-L), and high-dose HLD group (DSS + HLD-H). According to related studies [5, 19] and our previous work, all groups except the normal group were given 2.5% DSS ad libitum for seven days to replicate the ulcerative colitis model. The success of the model replication was based on a significant decrease in body weight, a significant increase in the disease activity index, a significant increase in serum inflammatory factor levels, and a significant shortening of colon length with a large amount of inflammatory infiltration. All mice were anesthetized with isoflurane, an inhalational anesthetic. The mice in the control group were not specially treated, and the mice in the DSS group were administered an equal amount of distilled water enema. The positive drug group was given 0.82 g/kg Mesalazine daily, the HLD-L group was given 12.81 g/kg/d HLD enema, and the HLD-H group was given 25.62 g/kg/d HLD enema for seven consecutive days. After the final administration, all mice fasted without water for 12 h. Blood was collected from the orbital plexus under anesthesia, centrifuged (4 °C, 600 × *g*, 10 min) to extract the supernatant, and stored at -80 °C. After blood collection, the mice were euthanized via inhalation of carbon dioxide in a specific device, and the colonic tissue was separated. A portion of the tissue from the distal colon was cut and fixed in a 4% paraformaldehyde solution, and the rest of the tissue was stored at -80 °C for subsequent experiments.

#### Disease activity index (DAI) scoring

Weight loss, stool consistency, and degree of intestinal bleeding were recorded daily in mice, and the DAI score was used to assess disease activity [19]. The DAI = (body weight loss score + stool consistency score + stool occult blood score) divided by 3.

#### Histological assessment of the colon

Colon tissues were fixed with 4% paraformaldehyde solution, dehydrated using gradient concentrations of ethanol and xylene, paraffin-embedded, serially sectioned at 4 μm, dewaxed, subjected to hematoxylin and eosin (HE) staining according to the manufacturer’s instructions, and subsequently placed under a microscope and photographed to observe the degree of inflammatory infiltration and colonic mucosal damage and to obtain a histological score [20].

#### Immunohistochemistry

Paraffin sections of preserved colon tissue were dewaxed and repaired, 3% hydrogen peroxide was added dropwise, incubated for 10 min, and then washed three times in phosphate buffer saline (PBS). Thereafter, TLR4, MyD88, and NF-κBp65 primary antibodies (1:200) were added in a dropwise manner after blocking with 5% bovine serum albumin (BSA). Samples were then incubated overnight and washed three times in PBS. Next, a secondary antibody was added and incubated for 1 h, 3,3'-Diaminobenzidine (DAB) solution and hematoxylin was added and re-stained, dehydrated, and sealed, and the sections were analyzed using microscopic analysis. Five fields were selected for each section and analyzed after quantitative grayscale scanning using an image analysis system. The expression of TLR4, MyD88, and NF-κBp65, and their localization in the cytoplasm and nucleus in the colon tissue sections were detected by immunohistochemistry.

#### ELISA for detection of cytokine levels in mouse serum

The levels of TNF-α, IL-6, IL-1β, and IL-10 in the serum were measured according to the manufacturer's instructions.

#### Detection of TLR4, MyD88, and NF-κB mRNA expression in mouse colon tissue

The mRNA expressions of TLR4, MyD88, and NF-κB in mice colon tissues, which are key genes in the TLR4/MyD88/NF-κB signaling pathway, were detected by Real-time PCR. Total RNA was extracted from intestinal tissues by the TRIzol method. cDNA was synthesized by reverse transcription. The primers were synthesized by GenScript Biotech Corporation (Nanjing, China) (Primer sequences were shown in Table 2). PCR was performed on an ABI 7500 Fast Real-Time PCR System (Applied Biosystems, Waltham, MA) in a volume of 10 μL. The reaction conditions were as follows: denaturation at 95 ℃ for 10 s. 30 s, denaturation at 95 ℃ for 5 s, annealing at 60 ℃ for 31 s, 40 cycles, and amplification at 95 ℃ for 15 s. Cycling thresholds (ΔCq) of the genes were normalized to the GAPDH and analyzed using the 2^−ΔΔCt^ method. Each experiment was repeated three times.

**Table 2:** Primer sequence list

Gene name	Primer sequence
TLR4	F:CCGCTCTGGCATCATCTTCA
	R:CCCACTCGAGGTAGGTGTTTCTG
MyD88	F:TATACCAACCCTTGCACCAAGTC
	R:TCAGGCTCCAAGTCAGCTCATC
NF-κB	F:TGACGGGAGGGGAAGAAATC
	R:TGAACAAACACGGAAGCTGG
GAPDH	F:GGCACAGTCAAGGCTGAGAATG
	R:ATGGTGGTGAAGACGCCAGTA

#### Detection of TLR4, MyD88, and NF-κBp65 expression in colon tissues using western blot

Colon tissue nuclear proteins were extracted from fresh colon tissues, quantified using the bicinchoninic acid (BCA) kit, and electrophoresed on 10% sodium dodecyl sulfate–polyacrylamide gels with 30 μg protein per well, and then transferred onto Polyvinylidene fluoride (PVDF) membranes and blocked for 2 h using a 5% skim milk powder solution. The corresponding primary antibody (1:1000) was added and incubated (overnight at 4 °C), and IgG-HRP (1:10,000) was added for 2 h. Proteins were detected via electrochemiluminescence using the ChemiDoc™ imaging system (Bio-Rad, CA, USA), followed by fractional value analysis to calculate the relative expression levels of individual protein bands.

#### Statistical processing methods

GraphPad Prism (version 9.0.0, CA, USA) was used to process the data, and all data were expressed as at least three replicates as mean ± standard deviation (x ± s). Means between multiple groups were analyzed using a one-way analysis of variance (one-way ANOVA), and the Student's *t*-test was used to compare the two groups. Differences were considered statistically significant at *P* < 0.05.

## Results

### Identification of UC targets

**Fig. 1:**
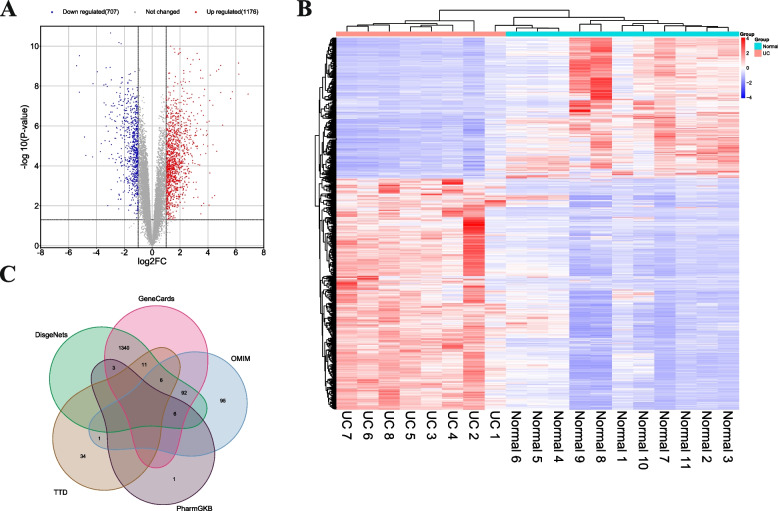
Targets related to UC. **A** DEGs in UC patients and healthy humans in volcano map; **B** Heat map of gene expression in UC patients and healthy humans; **C** UC-related genes acquired from series databases

The GSE10191 dataset was downloaded from the GEO database, and differential gene analysis was performed between UC patients and healthy individuals at |log_2_FC|> 1 and *P* < 0.05. The results are shown in the volcano and heat maps (Fig. 1 A and B). Finally, 1176 upregulated genes and 707 downregulated genes were identified in UC tissues compared to those in normal colon tissues.

Targets of UC-related genes were collected from GeneCards, PharmGKB, Therapeutic Target Database (TTD), DisgeNets, and Online Mendelian Inheritance in Man (OMIM) databases, including GeneCards with 1458 genes, 10 genes in PharmGkb, and 52 genes in TTD, with 1458 in DisgeNets and 200 in OMIM using “ulcerative colitis” as keywords. A total of 1589 relevant UC target genes were brought to the node after collection (Fig. 1C).

### UPLC-MS characterization of the chemical composition of HLD

**Fig. 2:**
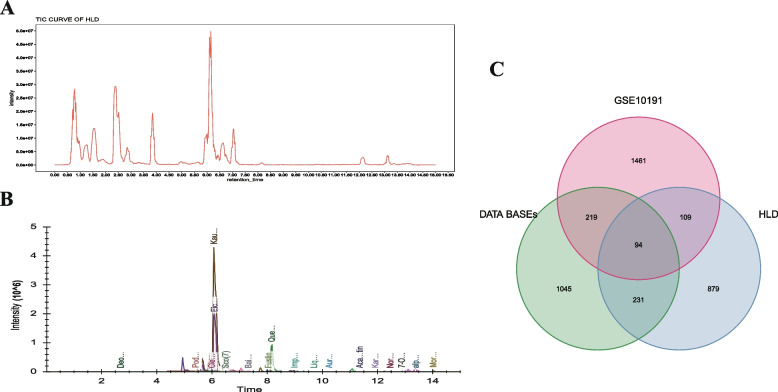
Targets of HLD related to UC. **A** TIC chromatograph of HLD; **B** Extracted ion chromatograph of chemicals in HLD related to UC; **C** Targets of HLD affected UC

The major compounds in HLD extracts were characterized using Sciex 6500 plus QTRAP UPLC-MS/MS. Figure 2A shows the total ion chromatograms (TIC). The major chemical components with peak areas greater than 7,000 were screened as candidate compounds. Compounds with OB > 30% and DL > 0.18 were selected and compared to the candidate compound ADME database, and a total of 57 compounds were identified and Exacted peak chromatograms were shown in Fig. 2B. Chemicals include 31 flavonoids, 7 terpenes, 6 steroids, 3 alkaloids, 2 lignans, 2 delsprays, 2 quinones, 2 coumarins, and 2 other compounds. The sample mass spectrometry data were imported into Skyline software, and the ion chromatograms of the 57 compounds were extracted, as shown in Fig. 2B. The chemical compositions were derived from reported literature data for known chemical components and based on their mass spectra and fragment ion properties, as shown in Table S1. In total, 1313 corresponding compound targets were obtained from the Swiss Target Prediction and PubChem databases.

A total of 1313 HLD-related targets, 1589 UC-related targets, and 1883 related targets from GSE10191 were screened for interactions using Venny, and 94 overlapping targets were identified as potential targets for HLD treatment of UC (Fig. 2C).

### PPI network construction

**Fig. 3:**
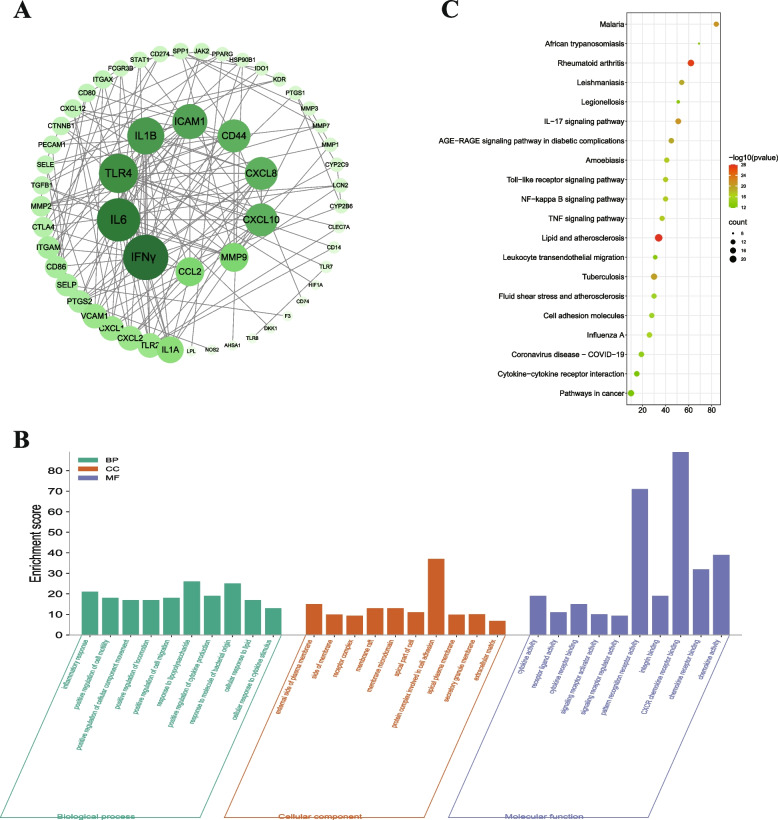
PPI, GO, and KEGG analysis of target genes. **A** PPI network construction; **B** GO enrichment analysis of target genes; **C** KEGG enrichment analysis of target genes

Cross-target genes between UC and HLD were imported into the STRING database for topological analysis. In the PPI network constructed using Cytoscape 3.8.2, 138 nodes and 488 edges were identified. The larger the number of nodes in the PPI network, the darker the color, indicating more interactions between the node proteins and surrounding proteins (Fig. 3A). The average DC, BC, CC value of the network was 5.75, 101.6 and 0.36 separately. Based on the topological analysis, six core targets were further screened:INF-γ, IL6, Toll-like receptor 4 (TLR4), IL1β, intercellular cell adhesion molecule-1(ICAM-1), CD44 (CD44), and chemokine (C-X-C pattern) ligand 8 (CXCL8).

### GO and KEGG enrichment

To elucidate the function and enrichment pathways of potential anti-UC genes in HLD, GO, and KEGG pathway enrichment analyses were performed on 94 overlapping targets based on the MetaScape platform, including biological process (BP), cellular component (CC), and molecular function (MF). A total of 3963 statistically significant GO terms were obtained in this study, including 3528 for BP, 112 for CC, and 323 for MF. Figure 3B shows the top 20 most significantly enriched terms for BP, CC, and MF. The GO enrichment results showed that the biological processes of the cross-targets were mainly enriched in the inflammatory response, positive regulation of cell motility, and positive regulation of CC motility. The CC analysis revealed that the therapeutic targets were mainly concentrated in the outer membrane side of the plasma membrane and the receptor complex. The MFs of the targets were enriched in cytokine activity, receptor-ligand activity, and cytokine receptor binding. The results suggest that the effects of HLD in UC treatment are mainly distributed on the cell membrane surface and are involved in intercellular signaling and regulation of cytokine synthesis and secretion.

KEGG pathway analysis was used to explore the functions and signaling pathways of the identified HLD anti-UC targets. We identified 105 statistically significant HLD-UC-related pathways (Fig. 3C), of which the top 20 pathways with significantly enriched gene numbers are presented as bubble plots. They mainly include IL-17, Toll-like receptor, NF-κB, and TNF signaling pathways. These results may provide key pathways through which HLD counteracts the effects of UC.

### C-P–T network construction

**Fig. 4:**
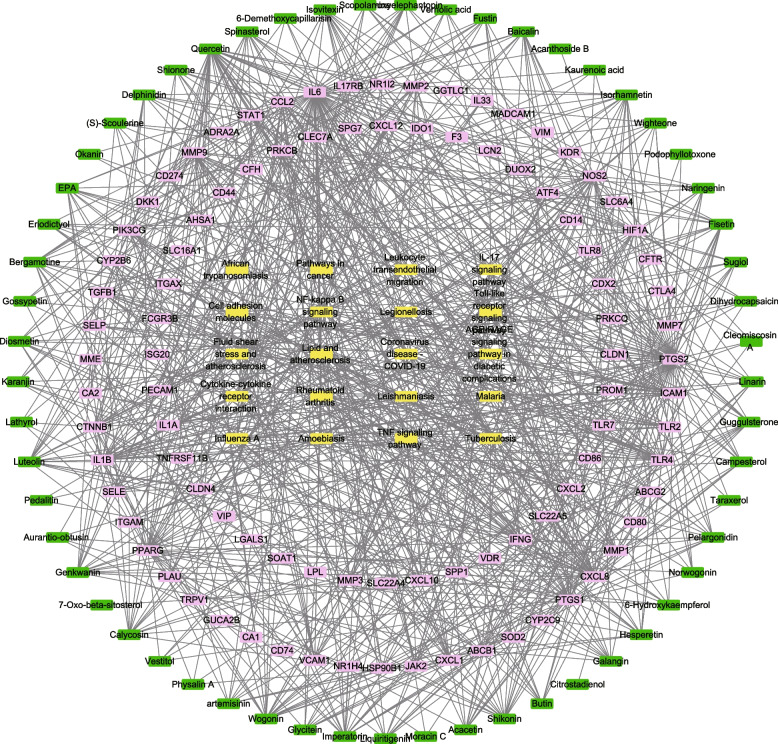
Chemicals-targets-pathways network of HLD on UC

In addition, a C-P–T network with 483 nodes and 3404 edges was constructed to elucidate the interrelationships among components, targets, and the first 20 pathways (Fig. 4). Together, these results suggest that HLD acts on UC through multiple pathways, targets, and a full range of cooperation.

### Molecular docking

**Fig. 5:**
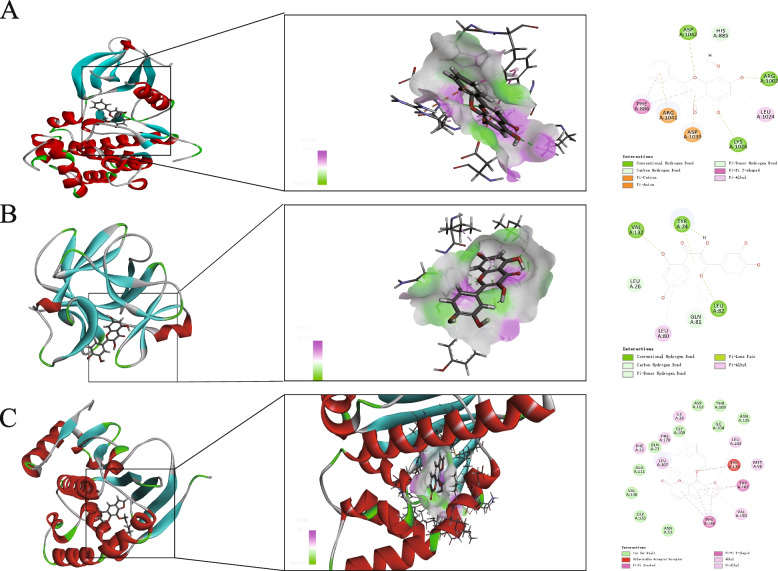
Molecular docking of key chemicals on hub genes of UC. **A** wogonin interacted with IL-6; **B** quercetin interacted with IL1β; **C** imperatorin interacted with TLR-4

Five active components (Quercetin, Wogonin, Shikonin, Luteolin, and Imperatorin) and six candidate target proteins (IL6, IL-1β, CXCL-8, INF-γ, TLR-4, and CD44) were docked using network analysis. The interaction of HLD active components with potential UC-related target genes was identified at the molecular level. The results showed that wogonin interacted with IL-6, quercetin interacted with IL1β, and imperatorin interacted with TLR-4 were shown in Fig. 5A–C. The imperatorin structure interacted with Phe 138 Trp 162 in π-conjugation, with Leu 107, Val 150, and Phe 22, 170 in alkyl conjugation, and with Gly135, Ala 111 with van der Waals.

Binding energy is often used to evaluate the affinity of a component for a protein target. It is generally accepted that a binding energy of less than—7.0 kcal/mol indicates a strong binding activity of the ligand to the receptor. The lower the binding energy, the higher the affinity of the receptor for the ligand and the more stable the conformation. These components could bind well to the active site of the protein target. Among them, IL6 had the lowest docking binding energy with Quercetin (- 9.0 kcal/mol) and JUN had the highest docking binding energy with nobiletin (- 5.4 kcal/mol), with an average binding energy of—6.96 kcal/mol, indicating that all five candidate compounds have good or even strong molecular docking with each candidate protein target. The five representative compounds of HLD (Quercetin, Wogonin, Shikonin, Luteolin, and Imperatorin) could bind well to the six core targets of UC (IL6, IL-1β, CXCL-8, INF-γ, TLR-4, and CD44) and may play a key role in UC treatment.

### Experimental validation

#### HLD ameliorates ulcerative colitis in mice induced by DSS

**Fig. 6:**
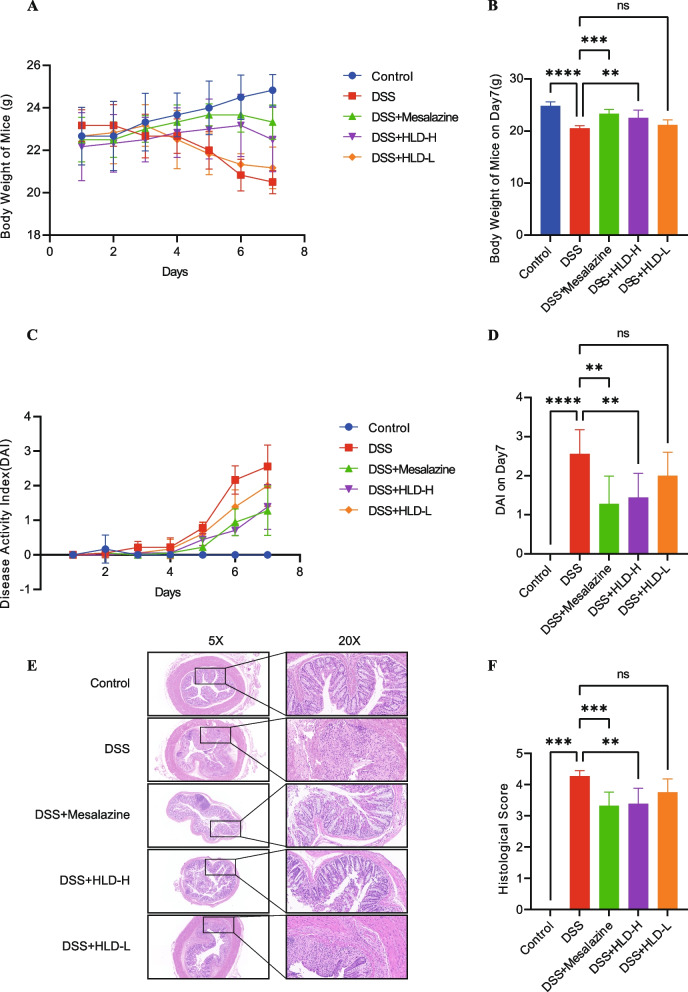
Effects of HLD on DSS-induced mice. **A**,** B** The trend of body weight changes in mice. **C**,** D** The trend of DAI changes in mice and a comparison of DAI. **E**,** F** HE staining results and histological scores of mice colonic tissues. All data are expressed as the mean ± SD. **p < 0.01, ***p < 0.001, ****p < 0.0001

To investigate the protective effect of HLD on acute UC, a mouse model of acute UC was established using 2.5% DSS administered for seven days. Mesalazine was used as a positive control in this study. The therapeutic effects of HLD on UC were initially evaluated in terms of two aspects: body weight changes and DAI scores in mice. From the third day, the body weight of the DSS-induced mice showed a significant and sustained decline. From day five, the DSS model mice exhibited symptoms such as diarrhea and blood in the stool. The DAI was significantly higher in the experimental group than in the control group. Compared with the DSS group, mice treated with high doses of HLD and mesalazine showed reduced weight loss induced by DSS (Fig. 6A and B).

DAI scores were considerably higher in the DSS group compared to the control group but appeared notably lower in both mesalazine and HLD-H groups than in the DSS group (Fig. 6C and D). In conclusion, our results suggest that HLD produces a protective effect in UC.

#### Effect of HLD on histopathological changes in the colon

HE staining was used to observe the intestinal histopathological changes in each group of mice. Colonic tissue appeared normal in structure and without ulcers, the intestinal glands were normal and well arranged, the mucosal muscle tissue was normal, and there was no lymphocyte infiltration in the control group (Fig. 6E). In the colonic tissue of DSS-treated mice, we observed severe necrosis and loss of mucosal epithelial cells, disappearance of intestinal glands, and massive infiltration of lymphocytes into the lamina propria and submucosa. In the mesalazine and HLD groups, the colonic structure was generally intact and well-arranged. Lymphocyte infiltration in the intrinsic and submucosal layers was reduced. In addition, we found that the colonic pathological features gradually reduced with increasing doses of HLD, and high-dose HLD and mesalazine treatment were superior to the HLD-L group. The Simplified Geboes Score was performed on HE images, and the results showed that HLD significantly reduced the Simplified Geboes Score in the DSS-induced UC model (Fig. 6F).

#### HLD inhibits inflammation levels in the serum of UC mice

**Fig. 7:**
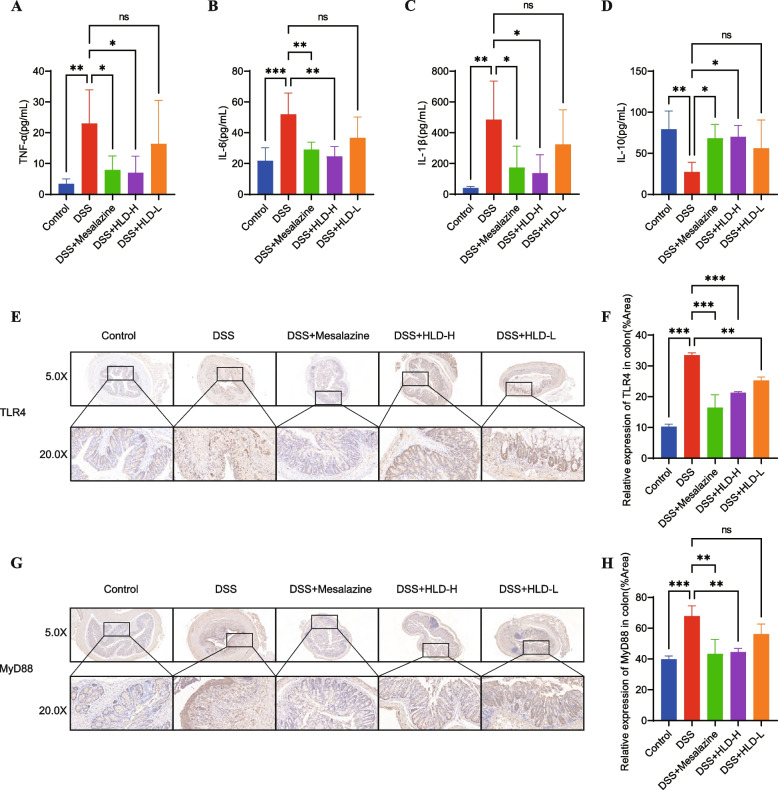
HLD suppressed inflammation in UC by suppressing TLR4 and MyD88. **A**-**D** Pro-inflammatory cytokines such as TNF-α, IL-6, and IL-1β and anti-inflammatory factors such as IL-10 levels were detected by ELISA. **E-H** Immunohistochemical sections and data comparison of TLR4 and MyD88 in mice colonic tissues. All data were expressed as the mean ± SD. *p < 0.05, **p < 0.01, and ***p < 0.001

Since pro-inflammatory factors play an important role in the pathogenesis and progression of UC, we investigated whether HLD has an anti-inflammatory effect on UC. Serum inflammatory factors were detected using ELISA. DSS notably promoted TNF-α, IL-1β, and IL-6 expression in the serum compared with the control group, while inhibiting the expression of IL-10 (Fig. 7A-D). Compared with the DSS group, HLD significantly inhibited TNF-α, IL-1β, and IL-6 levels and increased the IL-10 levels. These data suggest that HLD can inhibit the inflammatory response in DSS-induced UC.

#### HLD ameliorates DSS-induced UC via the TLR4/MyD88/NF-κB signaling pathway

According to numerous studies and network pharmacology analyses, inflammation plays a crucial role in the pathogenesis and course of UC, and the inflammatory response may be mediated in part by the TLR4/MyD88/NF-κB signaling pathway. Therefore, we investigated whether HLD could inhibit inflammation by regulating the expression of TLR4, MyD88, and p-NF-κB, and thus, have a therapeutic effect on UC.

We detected the expression of TLR4 and MyD88 in colonic tissues using immunohistochemistry. TLR4 and MyD88 expression levels were elevated in the DSS group (Fig. 7E-H). HLD decreased TLR4 and MyD88 expression and inhibited TLR4/MyD88 expression. Based on the above results, HLD exerts therapeutic effects by inhibiting the TLR4/MyD88 pathway.

**Fig. 8:**
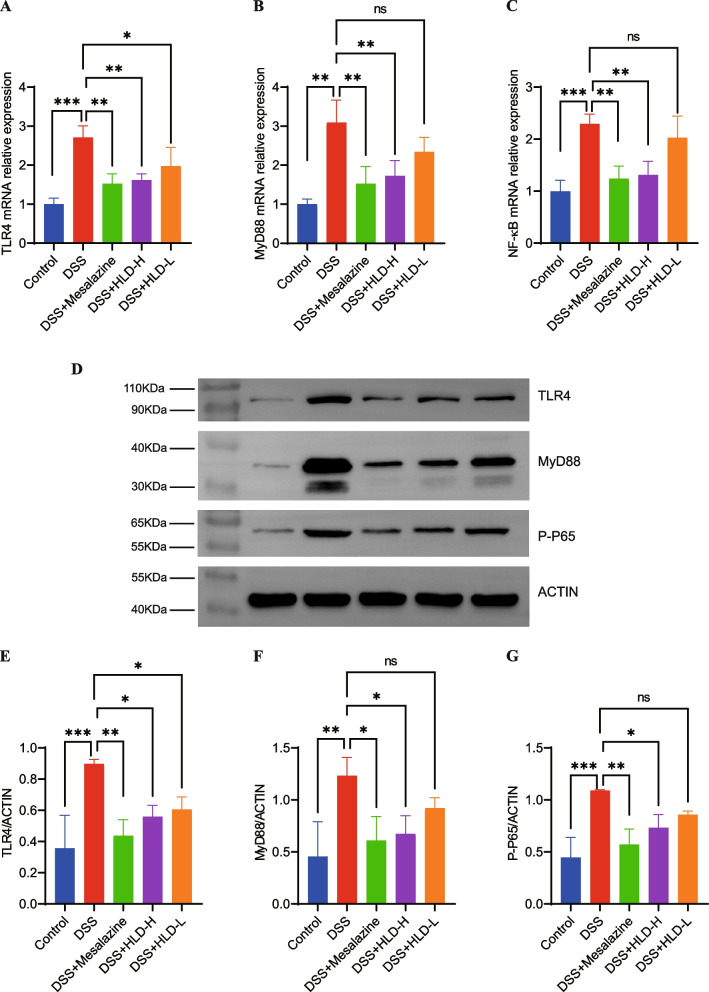
HLD suppressed inflammation via TLR4/MyD88/NF-κB pathway. **A-C** RT-PCR analysis of TLR4, MyD88, NF-κB mRNA expression in colon tissues. **D**-**G** Western blot analysis of TLR4, MyD88, NF-κB p-65 expression in mice colon tissues. Full-length blots/gels are presented in Supplementary materials-uncropped western blots. The western blots shown in the manuscript and the corresponding uncropped gels/blots in the supplementary material were linked. All data were expressed as the mean ± SD. *p < 0.05, **p < 0.01, and ***p < 0.001

Activation of the TLR4/MyD88 pathway can affect downstream NF-κB expression, thereby promoting inflammatory responses in UC patients. We investigated the effect of HLD-mediated TLR4/MyD88/NF-κB inhibition on the signaling effect using RT-PCR and western blot. The mRNA results indicated that TLR4, MyD88, and NF-κB mRNA expression increased significantly in UC model while compared with control group. While treated with HLD, the mRNA expression of TLR4, MyD88, and NF-κB reduced notably(Fig. 8A-C).

Western blotting results showed that DSS induced a significant increase in TLR4, MyD88, and p-NF-κB protein expression in colonic tissues (Fig. 8D-G), suggesting that the TLR4/MyD88/NF-κB signaling pathway was activated in UC. HLD treatment significantly downregulated TLR4, MyD88, and p-NF-κB protein levels. Similarly, mesalazine downregulated the protein expression levels of TLR4, MyD88, and p-NF-κB. These results suggest that HLD combats UC, in part, by inhibiting activation of the TLR4/MyD88/NF-κB signaling pathway.

## Discussion

Network pharmacology is a systems biology and biological network equilibrium approach to understanding drug-organism interactions and guiding new drug discovery [21, 22]. The holistic and systematic nature of network pharmacology aligns with the holistic diagnosis and treatment principles of Traditional Chinese Medicine (TCM), making it a popular approach for the study of Chinese herbal medicines [18, 23, 24]. Bioinformatics can provide insight into the potential modes of action and disease mechanisms at a molecular level [25].

In this study, we used a combination of network pharmacology, bioinformatics, and molecular docking to investigate the potential molecular mechanisms underlying HLD action in UC treatment. The results showed that pathways closely related to anti-UC effects of HLD included IL-17, Toll-like receptor, NF-κB, and TNF signaling pathways. In the PPI networks, IFN-γ, IL6, TLR4, IL1β, ICAM1, CD44, CXCL-8, CXCL-10, and MMP9 displayed the strongest correlation with the anti-UC activity of HLD.

Immunological studies have confirmed that immune cells and factors contributing to the pathogenesis of UC include IL-6, TNF-α, IL-17, IFN-γ, IL-21, IL-13, IL-4, and IL-5 [26]. Currently, the treatment of UC focuses on targeting TNF-α, IL-12, and IL-23, which are among the cytokines known to contribute to the pathology of the disease. Infliximab and adalimumab are therapeutic agents for severe UC and are targeted inhibitors of TNF-α [2, 27, 28]. Ustekinumab, an IL-12/IL-23 inhibitor, is an emerging drug for the treatment of severe UC [2, 28]. The top five target genes and four major signaling pathways enriched by HLD for anti-UC treatment have been found to include traditional pro-inflammatory cytokines, such as IFN-γ, IL-6, and IL-1β, which play crucial roles in various inflammatory responses. IL-6 is widely recognized as a cytokine associated with inflammation. In the present study, the ELISA results confirmed that the levels of IL-6, TNF-α, and IL-1β were significantly increased in the colonic tissues of DSS mice, but their expression was reduced in the Mesalazine and HLD-H groups. Cytokine IL-8, also known as CXCL8, is produced by macrophages and epithelial cells. It acts as a chemotactic factor for neutrophils, which plays a crucial role in IBD development [29]. IL-8 promotes the migration of neutrophils to the intestine, where it releases inducible nitric oxide synthase and matrix metalloproteases that cause damage to epithelial cells, which is a hallmark of IBD [26]. Inflammatory epithelial cells, macrophages, T lymphocytes, and neutrophils from patients with UC have been found to produce several chemokines, including IL-8 [29]. NF-κB is essential for the induction of IL-8 expression [29]. The NF-κB and IL-17 signaling pathways are classical inflammatory pathways, and our previous studies demonstrated that HLD exerts anti-inflammatory effects by inhibiting these two signaling pathways [13]. IFN-γ is the sole type II interferon secreted by CD4 and CD8 cytotoxic T cells during antigen-specific immune responses. Aberrant expression of IFN-γ is associated with numerous autoimmune diseases. CD4 + T cells release inflammatory cytokines such as IFN-γ and IL-15, which in turn promote the transformation of intraepithelial lymphocytes into cytotoxic CD8 + T cells. These cytotoxic CD8 + T cells subsequently target and eliminate intestinal epithelial cells, disrupting the mucosal barrier [30]. Upon activation, IFN-γ recruits and phosphorylates signal transducer and activator of transcription 1(STAT1). Phosphorylated STAT1 forms dimers and translocates to the nucleus, binding to the Gamma-activated sequence (GAS) in the promoter region of the genome, thereby regulating downstream gene transcription [31]. The IFN-γ/STAT1 signaling pathway can activate the MAPK, PI3K-Akt, and NF-κB signaling pathways [31].

The TLR family in mammals consists of 13 members, and these receptors are responsible for recognizing specific patterns of microbial components known as pathogen-associated molecular patterns (PAMPs) [32, 33]. TLRs recognize their ligands and each TLR recognizes different parts of PAMPs. All TLRs, except TLR3, activate the MyD88-dependent signaling pathway, which leads to the production of pro-inflammatory cytokines [34]. The MyD88 signaling pathway is triggered when the activated Toll/IL-1R homologous region domain of the TLR complex associates with MyD88, which recruits interleukin-1 receptor-associated kinase (IRAK)1, IRAK4, and TNF receptor-associated factor 6(TRAF6). The recruitment of these components results in the phosphorylation of IRAK1 and TRAF6, which propagates the signal to the downstream MAP kinase-AP1 and IKK complex, NF-κB [35]. This activates transcription factors and leads to the activation of NF-κB, an important regulator of inflammation. NF-κB is capable of regulating the transcription and expression of TNF-α, IL-1β, and IL-6, as well as other inflammatory mediators and growth factors. The expression of these factors by NF-κB further sustains and activates NF-κB, thereby exacerbating inflammatory injury [32].

In this study, the TLR signaling pathway, NF-κB signaling pathway, and TLR4 were all closely associated with anti-UC HLD using network pharmacology and bioinformatics analysis. Our previous study demonstrated that HLD alleviates UC in mice by inhibiting the NF-κB signaling pathway [14]. *TLR4* is an upstream gene in the NF-κB signaling pathway. Therefore, this study focused on the mechanism of UC alleviation by HLD via the TLR4/MyD88/NF-κB pathway.

TLRs are key factors in the innate immune system. After TLRs are activated, they can transmit inflammatory reaction information through the MyD88-dependent signal pathway, mediate the expression and release of inflammatory factors, and produce inflammatory injury. This study results indicated that HLD-H was effective in decreasing the DAI and colonic histological scores, thereby alleviating the symptoms of diarrhea and rectal bleeding caused by DSS. Additionally, HLD-H reduced the expression of TLR4, MyD88, and NF-κB, and lowered the levels of pro-inflammatory cytokines TNF-α, IL-1β, and IL-6 in the colonic tissue of mice with DSS-induced UC. In conclusion, this study suggests that HLD-H may have therapeutic potential for UC treatment by inhibiting the TLR4/MyD88/NF-κB signaling pathway, which is closely associated with UC development [32, 33, 36].

## Conclusions

This study utilized network pharmacology and bioinformatics analysis to analyze the active ingredients of HLD aqueous solution and explore its multi-target mechanism for treating UC. In addition, the study validated that HLD exerts an anti-UC effect in a DSS-induced UC mouse model by inhibiting the TLR4/MyD88/NF-κB pathway. The results provide theoretical evidence for the prevention and treatment of UC with HLD. However, the unknown active ingredients of HLD in the blood become a limitation of this study, which is a gap that will be addressed in future studies. Furthermore, the lack of in vitro experimental verification is another limitation, as the anti-UC effect of HLD was only validated through in vivo experiments inhibiting the TLR4/MyD88/NF-κB pathway. Therefore, our future research will focus on exploring the mechanism of HLD's anti-UC effect from a metabolomics perspective, combining network pharmacology and in vivo/in vitro experiments to provide evidence.

### Supplementary Information

Supplementary Material 1.

Supplementary Material 2.

## Data Availability

The data supporting the conclusions of this article are included within the article. The data used during the current study are available from the corresponding author upon reasonable request.

## Abbreviations

HLD: Huangkui Lianchang Decoction
TCM: Traditional Chinese Medicine
DSS: Dextran sulfate sodium
GEO: Gene expression omnibus
HE: Hematoxylin–eosin
HLD-L: Low-dose HLD
DAI: Disease activity index
MyD88: Myeloiddifferentiationfactor88
OMIM: Online Mendelian Inheritance in Man
IL-6: Interleukin 6
CXCL8: Chemokine ligand 8
KEGG: Kyoto Encyclopedia of Genes and Genomes
DEG: Differentially expressed genes
GO: Gene Ontology
UC: Ulcerative colitis
IBD: Inflammatory bowel disease
UPLC-MS/MS: Ultra-performance liquid chromatography-tandem mass spectrometry
TTD: Therapeutic Target Database
ELISA: Enzyme-linked immunosorbent assay
HLD-H: High-dose HLD
TLR4: Toll-like receptor 4
NF-κB: Nuclear factor kappa-B
TIC: Total ion chromatograms
IL-1β: Interleukin-1β
IFN-γ: Interferon-γ
STAT1: Signal transducer and activator of transcription 1
PDB: Protein Data Bank
SPF: Specific pathogen-free
