# Supplementary material for: Integrated network pharmacology and bioinformatics to identify therapeutic targets and molecular mechanisms of Huangkui Lianchang Decoction for ulcerative colitis treatment
Source: BMC Complement Med Ther. 2024 Jul 23;24:280. doi: 10.1186/s12906-024-04590-3 (PMC11267728; doi:10.1186/s12906-024-04590-3)
Supplement: Supplementary file 1 — Supplementary Material 1. [file 12906_2024_4590_MOESM1_ESM.docx]

Table S1 Chemicals related to UC detected in HLD by UPLC-MS/MS

| Chemical Name | Formula | CLASS | MS^1^ | MS^2^ | ionmode | Retention time（min） |
| --- | --- | --- | --- | --- | --- | --- |
| Kaurenoic acid | C_20_H_30_O_2_ | Diterpenoids | 303.2 | 257.2 | + | 5.9 |
| Quercetin | C_15_H_10_O_7_ | Flavonoids | 303 | 153 | + | 8.07 |
| Okanin | C_15_H_12_O_6_ | Chalcones | 289.1 | 135 | + | 5.99 |
| Delphinidin | C_15_H_10_O_7_ | Flavonoids | 303 | 229 | + | 6.86 |
| Linarin | C_28_H_32_O_14_ | Flavonoids | 593.2 | 285.1 | + | 7.65 |
| Galangin | C_15_H_10_O_5_ | flavonoids | 271.2 | 196.9 | + | 11.14 |
| Artemisinin | C_15_H_22_O_5_ | Sesquiterpenoids | 283.2 | 110.1 | + | 13.02 |
| Eicosapentaenoic acid | C_20_H_30_O_2_ | Fatty Acyls | 303.2 | 285.2 | + | 6.86 |
| Scoulerine | C_19_H_21_NO_4_ | Alkaloids | 328.2 | 268.1 | + | 5.77 |
| Luteolin | C_15_H_10_O_6_ | flavonoids | 286.9 | 240.9 | + | 8.03 |
| Naringenin | C_15_H_12_O_5_ | flavonoids | 273.3 | 152.8 | + | 8.86 |
| Eriodictyol | C_15_H_12_O_6_ | flavonoids | 288.9 | 163 | + | 7.9 |
| Isovitexin | C_21_H_20_O_10_ | Flavonoids | 433.1 | 415.1 | + | 5.85 |
| Campesterol | C_28_H_48_O | Steroids | 383.4 | 147.1 | + | 12.18 |
| Moracin C | C_19_H_18_O_4_ | Phenols | 311.1 | 55.1 | + | 13.99 |
| Hesperetin | C_16_H_1_4O_6_ | flavonoids | 303.3 | 178.9 | + | 9.2 |
| Fisetin | C_15_H_12_O_6_ | flavonoids | 286.9 | 241 | + | 7.03 |
| Demethoxycapillarisin | C_15_H_10_O_6_ | Flavonoids | 287.1 | 269 | + | 6.51 |
| Podophyllotoxinone | C_22_H_20_O_8_ | Lignans | 413.1 | 395.1 | + | 5.42 |
| Diosmetin | C_16_H_12_O_6_ | flavonoids | 301.3 | 285.8 | + | 9.15 |
| Pelargonidin | C_15_H_10_O_5_ | Flavonoids | 271.1 | 121 | + | 8.98 |
| Calycosin | C_16_H_12_O_5_ | Flavonoids | 285.1 | 267.1 | + | 9.1 |
| Gossypetin | C_15_H_10_O_8_ | Flavonoids | 319 | 109 | + | 6.46 |
| Lathyrol | C_20_H_30_O_4_ | Diterpenoids | 335.2 | 317.2 | + | 7.15 |
| Vestitol | C_16_H_16_O_4_ | Flavonoids | 273.1 | 123 | + | 6.51 |
| Karanjin | C_18_H_12_O_4_ | Flavonoids | 293.1 | 105 | + | 11.67 |
| Aurantio-obtusin | C_17_H_1_4O_7_ | Anthraquinones | 331.1 | 313.1 | + | 10.28 |
| Acanthoside B | C_28_H_36_O_13_ | Lignans | 581.2 | 419.2 | + | 6.51 |
| Wighteone | C_20_H_18_O_5_ | Flavonoids | 339.1 | 55.1 | + | 13.01 |
| Genkwanin | C_16_H_12_O_5_ | flavonoids | 284.9 | 269.9 | + | 11.1 |
| Isorhamnetin | C_16_H_12_O_7_ | flavonoids | 317.1 | 302.1 | + | 9.23 |
| 7-Oxo-beta-sitosterol | C_29_H_48_O_2_ | Steroids | 429.4 | 411.4 | + | 13.54 |
| Bergamotine | C_21_H_22_O_4_ | Coumarins | 339.2 | 203 | + | 13.03 |
| Wogonin | C_16_H_12_O_5_ | Flavonoids | 285.1 | 270.1 | + | 11.08 |
| Scopolamine | C_17_H_21_NO_4_ | Alkaloids | 304.2 | 138.1 | + | 6.86 |
| Pedalitin | C_16_H_12_O_7_ | Flavonoids | 317.1 | 109 | + | 12.08 |
| Shionone | C_30_H_50_O | Triterpenoids | 427.4 | 409.4 | + | 12 |
| Butin | C_15_H_12_O_5_ | Flavonoids | 273.1 | 121 | + | 8.83 |
| Dihydrocapsaicin | C_18_H_29_NO_3_ | Alkaloids | 308.2 | 137.1 | + | 11.94 |
| Physalin A | C_28_H_30_O_10_ | Steroids | 527.2 | 509.2 | + | 12.1 |
| Cleomiscosin A | C_20_H_18_O_8_ | Coumarins | 387.1 | 137.1 | + | 5.74 |
| 6-Hydroxykaempferol | C_15_H_10_O_7_ | Flavonoids | 303 | 105 | + | 6.84 |
| Norwogonin | C_15_H_10_O_5_ | Flavonoids | 271.1 | 145 | + | 11.83 |
| Shikonin | C_16_H_16_O_5_ | Quinones | 289.1 | 271.1 | + | 5.14 |
| Sugiol | C_20_H_28_O_2_ | Diterpenoids | 301.2 | 257.2 | + | 8.21 |
| Deoxyelephantopin | C_19_H_20_O_6_ | Sesquiterpenoids | 345.1 | 69 | + | 2.66 |
| Glycitein | C_16_H_12_O_5_ | flavonoids | 285.3 | 269.8 | + | 7.91 |
| Guggulsterone | C_22_H_30_O_3_ | Steroids | 343.2 | 325.2 | + | 11.08 |
| Citrostadienol | C_30_H_50_O | Steroids | 427.4 | 409.4 | + | 12.29 |
| Vernolic acid | C_18_H_32_O_3_ | Fatty Acyls | 297.2 | 279.2 | + | 11.88 |
| Imperatorin | C_16_H_1_4O_4_ | Phenylpropanoids | 271.1 | 203 | + | 8.89 |
| Acacetin | C_16_H_12_O_5_ | flavonoids | 285.3 | 269.8 | + | 11.03 |
| Baicalin | C_21_H_18_O_11_ | flavonoids | 447 | 430.9 | + | 7.26 |
| Liquiritigenin | C_15_H_12_O4 | Flavonoids | 257.1 | 121 | + | 9.65 |
| Fustin | C_15_H_12_O_6_ | Flavonoids | 289.1 | 121 | + | 7.59 |
| Taraxerol | C_30_H_50_O | Triterpenoids | 427.4 | 409.4 | + | 12.71 |
| Spinasterol | C_29_H_48_O | Steroids | 395.4 | 83.1 | + | 13.32 |
