# Supplementary material for: Integrated network pharmacology and bioinformatics to identify therapeutic targets and molecular mechanisms of Huangkui Lianchang Decoction for ulcerative colitis treatment
Source: BMC Complement Med Ther. 2024 Jul 23;24:280. doi: 10.1186/s12906-024-04590-3 (PMC11267728; doi:10.1186/s12906-024-04590-3)
Supplement: Supplementary file 2 — Supplementary Material 2. [file 12906_2024_4590_MOESM2_ESM.pptx]

## Slide 1
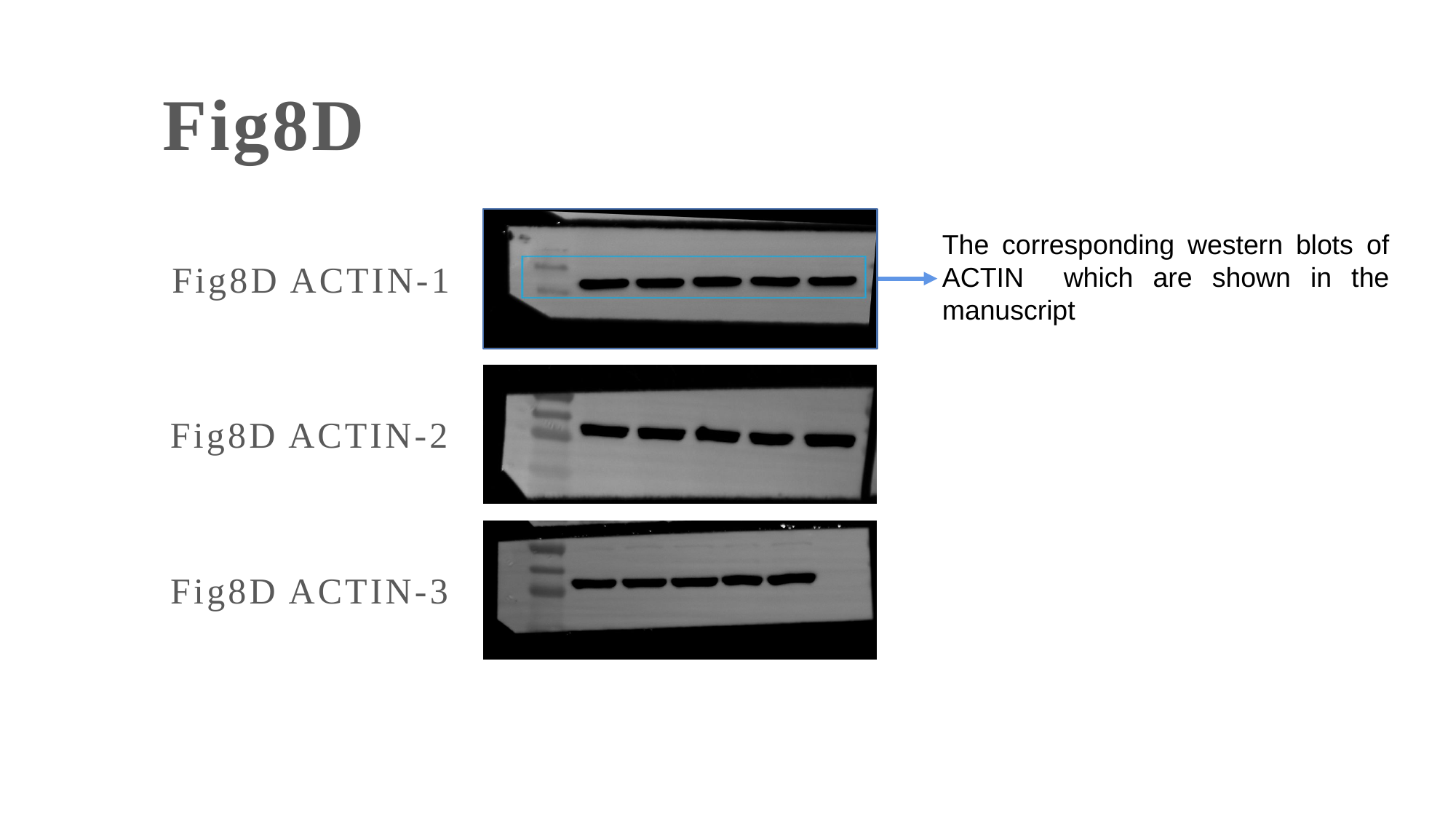

Fig8D
The corresponding western blots of ACTIN which are shown in the manuscript
Fig8D ACTIN-1
Fig8D ACTIN-2
Fig8D ACTIN-3

## Slide 2
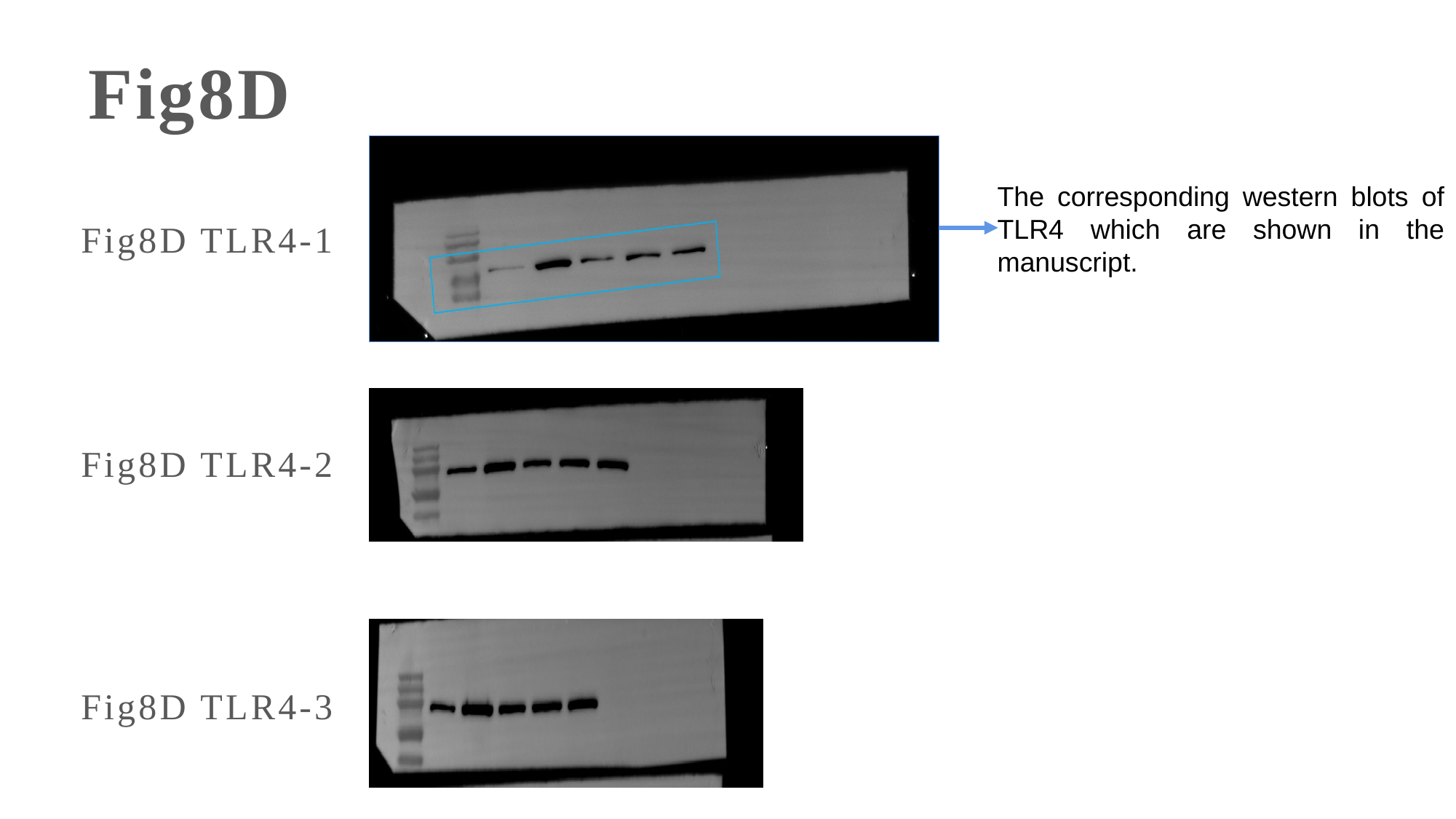

Fig8D
The corresponding western blots of TLR4 which are shown in the manuscript.
Fig8D TLR4-1
Fig8D TLR4-2
Fig8D TLR4-3

## Slide 3
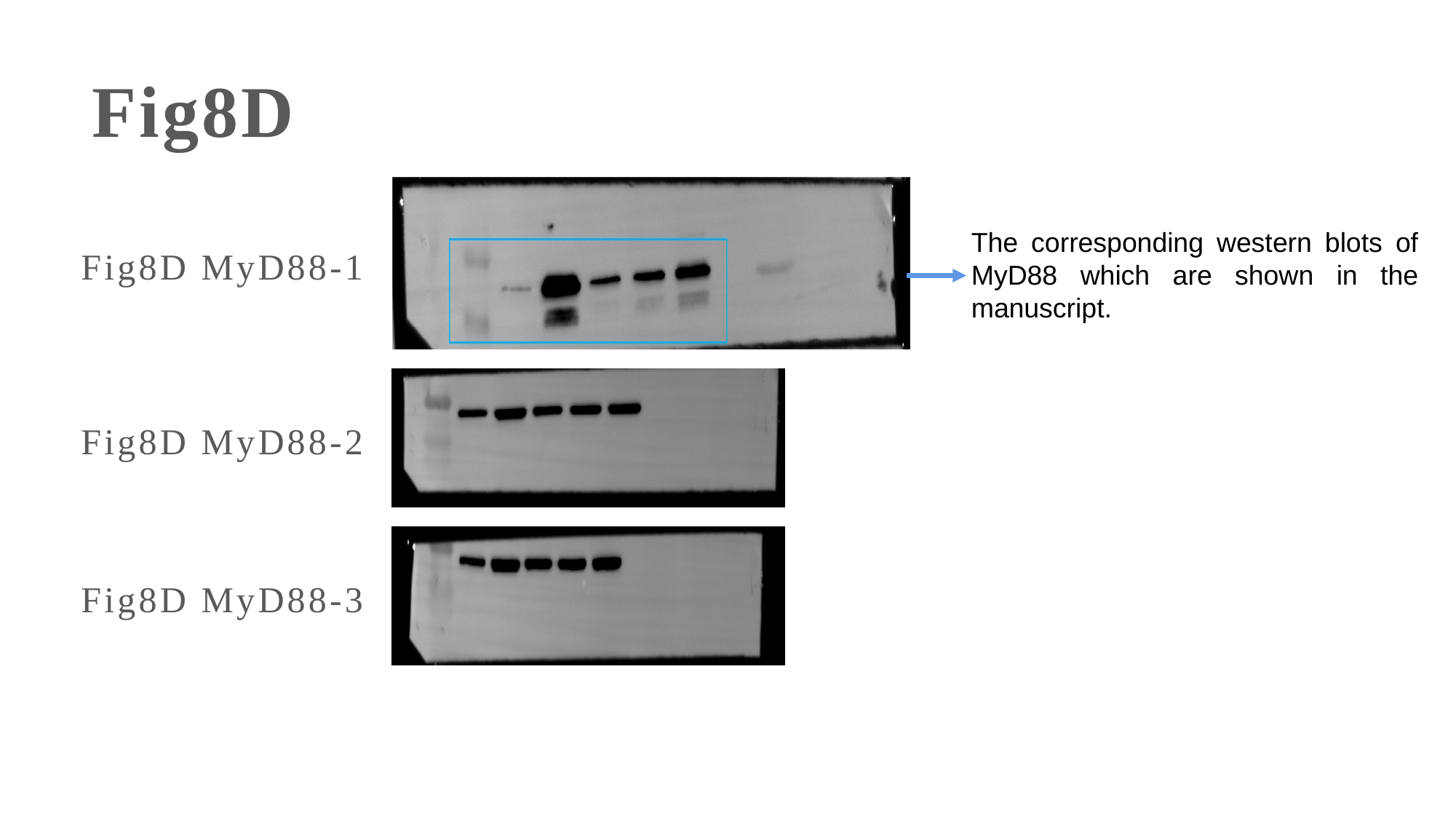

Fig8D
Fig8D MyD88-1
The corresponding western blots of MyD88 which are shown in the manuscript.
Fig8D MyD88-2
Fig8D MyD88-3

## Slide 4
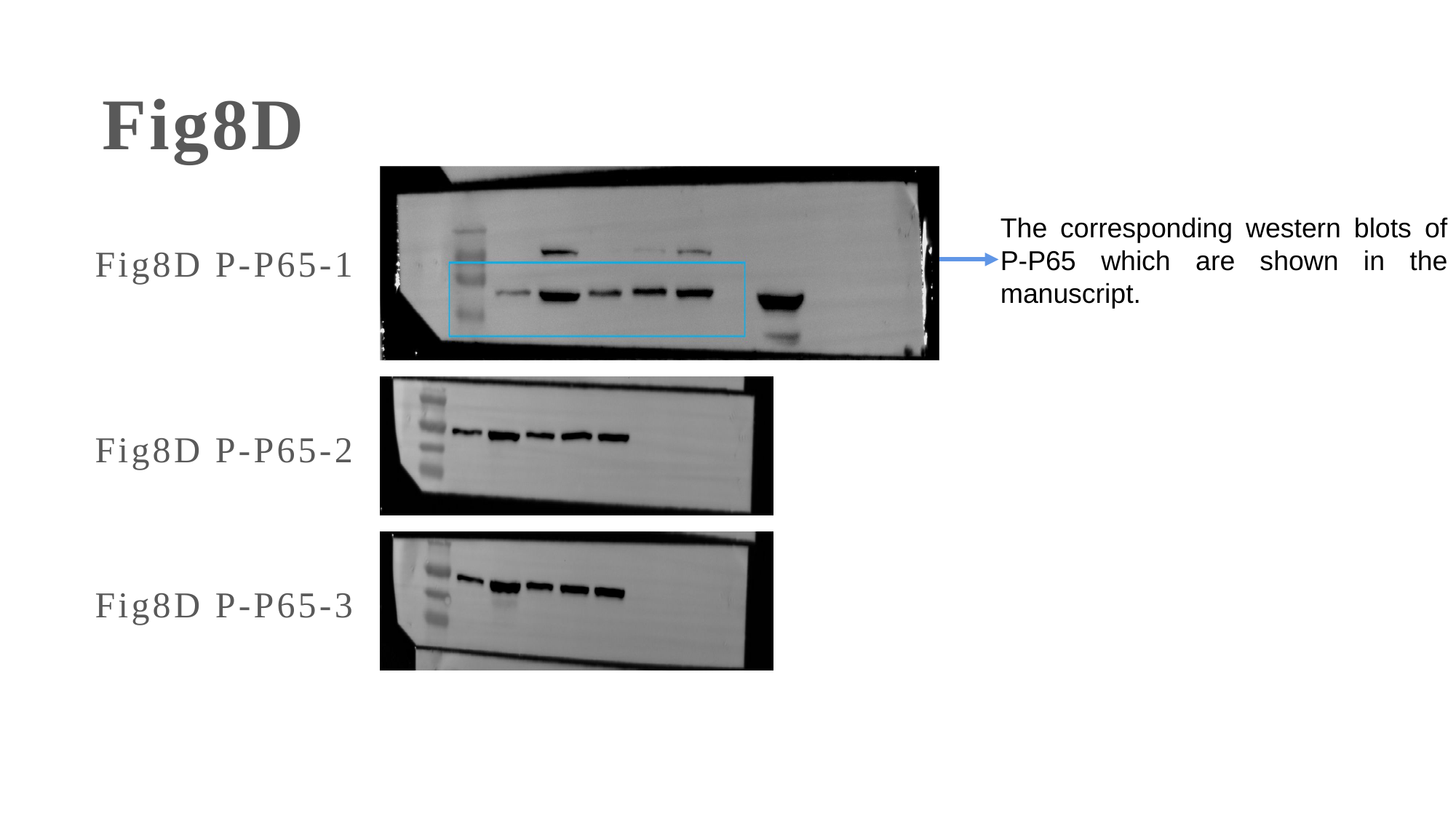

Fig8D
The corresponding western blots of P-P65 which are shown in the manuscript.
Fig8D P-P65-1
Fig8D P-P65-2
Fig8D P-P65-3
